# Supplementary material for: Chronic Exposure to High Fat Diet Affects the Synaptic Transmission That Regulates the Dopamine Release in the Nucleus Accumbens of Adolescent Male Rats
Source: Int J Mol Sci. 2023 Feb 28;24(5):4703. doi: 10.3390/ijms24054703 (PMC10003643; doi:10.3390/ijms24054703)
Supplement: Supplementary file 1 [file ijms-24-04703-s001.zip › ijms-1996503-supplementary.pdf]

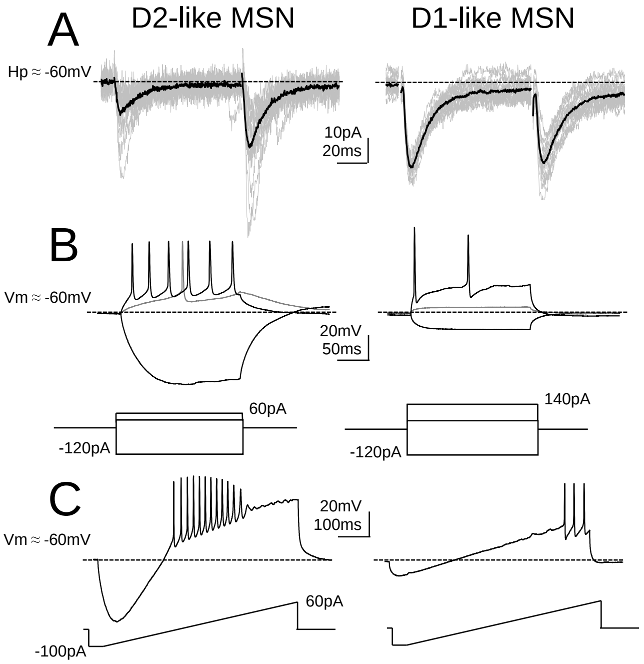

Supplemental Figure S1. Differential paired pulse ratio and firing properties recorded in two putative cells D1-like MSN and D2-like MSN of NAc core obtained in basal conditions in control male rats. (A) EPSCs evoked by paired-pulse stimulation at 80 ms interstimulus intervals, recorded at -60 mV of holding potential. Black traces represent the average of twenty consecutive stimulations 3 s apart for each cell. (B) Voltage responses obtained form same cells showed in (A) to injection of rectangular current pulses recorded at -60 mV at resting membrane

potential. (C) Voltage responses to ramp currents injected into the same two cells showed above.
